# Factors Influencing Early Readmission after Discharge against Medical Advice from the Emergency Department

**DOI:** 10.3390/healthcare9080986

**Published:** 2021-08-03

**Authors:** Byeong-Keon Moon, Ryeok Ahn, Deulle Min, JaeLan Shim

**Affiliations:** 1 Department of Emergency Medicine, University of Ulsan College of Medicine, Ulsan University Hospital, Ulsan 44033, Korea; mythofmbk@naver.com (B.-K.M.); amcer1010@uuh.ulsan.kr (R.A.); 2 Department of Nursing, College of Medicine, Wonkwang University, Iksan 54536, Korea; dlmin20@wku.ac.kr; 3 College of Nursing, Dongguk University, Gyeongju 38066, Korea

**Keywords:** emergency room, AMA discharge, early readmission, insurance

## Abstract

Discharge against medical advice (DAMA) and readmissions are important issues worldwide and can lead to adverse clinical outcomes, financial burden, and exposure of healthcare workers to unintended medical disputes. This study aimed to identify factors that affect readmissions within 48 h after DAMA. This retrospective study utilized the medical records of patients who visited an emergency medical center in Korea for treatment and were readmitted during a 10-year period. Factors predicting readmission after being DAMA were identified using logistic regression analysis. The total number of patients who were DAMA during the study period was 5445, of which 351 were readmitted to the emergency department within 48 h (6.4%). Factors influencing readmission included medical aid (odds ratio (OR) = 2.02, 95% confidence interval (CI): 1.46–2.83) and foreign worker insurance (OR = 2.07, 95% CI: 1.04–4.09) as their health insurance, as well as presenting for readmission by car (OR = 1.41, 95% CI: 1.08–1.82). Healthcare workers should treat patients who are DAMA and those who are likely to return with a more careful and preventative management strategy so that potential clinical, legal, and economic impacts of DAMA can be mitigated.

## 1. Introduction

Discharge against medical advice (DAMA) is when a patient chooses to leave the hospital before a physician advises discharge [1]. In addition, it is considered an escape (absence without leave, absconding, or elopement) when the patient leaves the hospital without notification from an involuntary unit or walks out of a voluntary unit. Some clinicians and researchers have considered it to be a form of DAMA. However, others do not regard escape as a form of DAMA because the essential element of a physician’s expressed advice against leaving in this situation is lacking [2]. The rate of DAMA in the emergency department (ED) is approximately 0.1–2.7% [3,4] and is increasing [5,6]. DAMA is a global issue and can lead to the death of the patient, re-hospitalization, exacerbation of latent diseases, and exposure of healthcare workers to unintended medical disputes [3,6]. The reasons for DAMA are numerous and include family obligations, prolonged waiting time, patient dissatisfaction, and the most significant of all, financial problems [3,4,7,8]. Studies have also identified predictors associated with DAMA, which include race/ethnicity, male sex, lack of health insurance, and history of alcohol or other substance abuse [3,9]. Due to the nature of emergency medicine, the relationship between the patient and the healthcare providers is established in a situation with a shortage of manpower, facilities, and overcrowding [10]. Dissatisfaction with medical services due to delays and costs often leads to DAMA [11]. In addition, it could be the result of symptom improvement after treatment administration in the ED [5].

Patients who have been DAMA are often more likely to return to the hospital because of the same or related issues [4,5]. They are also more than twice as likely to be readmitted within one month and more than three times as likely to return to the ED within a week compared to patients who were routinely discharged [5]. Therefore, the burden of medical expenses due to readmission is greater for patients who were DAMA [12]. Interestingly, despite these issues, few studies have examined the factors that contribute to readmission after DAMA, and only 0.39–27% included early ED readmission within 48 h to 90 days after discharge [13]. 

A better understanding of patients who are DAMA can lead to solutions that minimize their negative impact, including improving the continuity of care and access to community-based services. Moreover, strategies are needed to reduce the number of patients who refuse medical advice and minimize the risk and adverse outcomes by identifying predictive factors that can be recognized by healthcare providers. Thus, this study aimed to investigate the characteristics of patients who were DAMA from the ED and identify the factors that predict readmission within 48 h.

## 2. Materials and Methods

### 2.1. Study Design

This was a retrospective, descriptive research study that aimed to identify factors affecting hospital readmission of patients who were DAMA from the ED within 48 h. 

### 2.2. Data Collection and Procedure

Data were collected from the medical records of patients who were DAMA and readmitted to the ED within 48 h at a regional emergency medical center in Korea between 1 January 2010 and 31 December 2019 (10 years). Patients were identified as those with “AMA discharge” on their medical records, and unidentified cases and escape patients were excluded. Only patients who were readmitted to the hospital within 48 h were analyzed. Patients who were specified as “AMA discharge” but were not actually DAMA after checking their records were excluded, and no other variables were restricted. 

Normal disposition included hospitalization, discharge, or transfer. Death on arrival, death after arrival, and patients admitted for purposes other than medical treatment, such as obtaining copies of medical records and issuing documents, were classified as not having received treatment.

### 2.3. Ethical Consideration

This retrospective study was approved by the Institutional Review Board of the research institution (IRB No: 2020-04-018-001) and was conducted in accordance with the Declaration of Helsinki. The need for informed consent was waived due to the retrospective nature of the study.

### 2.4. Measurements

The collected variables included demographic information (gender, age, insurance type), type of transportation, length of stay in the ED, and the main diagnosis. The reason for DAMA was based on the physician’s notes, and nursing record details in the medical records. In the classification of symptoms of patients DAMA after their first emergency room visit, comprehensive classification is possible by coding not only the diagnosis but also the reason for visit, treatment, and clinical pathology examination. Classification was based on the International Classification of Primary Care (ICPC) [14].

### 2.5. Statistical Analysis 

Demographic information and major variables were presented using descriptive statistics. Categorical variables were expressed as percentages, and continuous ones as means and standard deviations. For the analysis, the Chi-squared test was used for categorical dependent variables and the student’s t-test for continuous variables. Logistic regression analysis was used to determine the factors influencing readmissions within 48 h after DAMA from the ED. The SPSS 24.0 program (IBM Corp. Armonk, NY, USA) was used for the statistical analysis, and the significance level was set at *p* < 0.05.

## 3. Results

### 3.1. General Characteristics 

**Figure 1:**
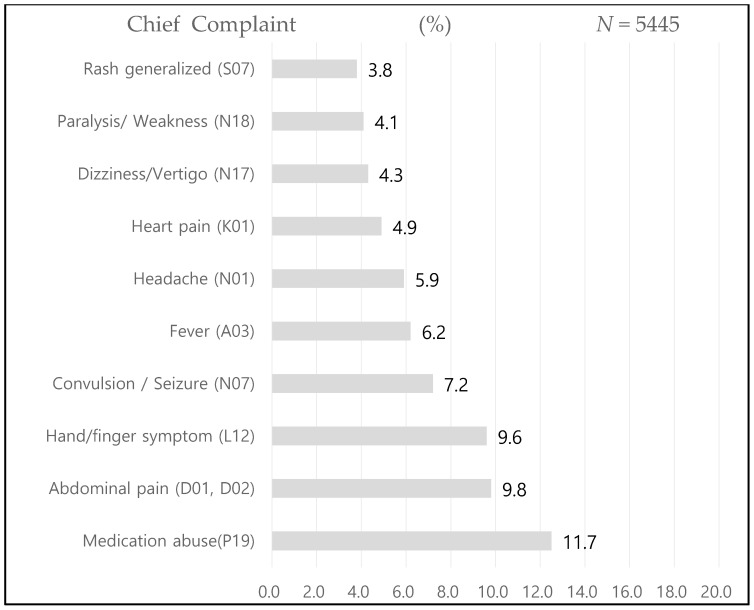
Distribution of the main chief complaints of patients DAMA after their first emergency room visit in this study.

The total number of patients DAMA from the ED was 5445, of which 351 returned within 48 h, accounting for 6.4% of the total number of patients. The average age of patients DAMA was 43.5 ± 22.1 years, and the age group with the most readmissions was 51–60 years (21.4%). In terms of sex, males accounted for more than half (57.5%) of patients who were readmitted to the ED, and males were more likely to be readmitted after DAMA than females (42.5%). There was no significant difference in age and sex between the two groups. Regarding insurance type, the national health insurance accounted for the largest percentage (78.3%), followed by medical aid (14.0%), industrial accident insurance for foreign workers (2.8%), and private insurance (1.7%). There was a significant difference between the two groups in terms of insurance type (χ^2^ = 26.23, *p* < 0.001) (Table 1). The main chief complaints in the 10 ranks of patients DAMA after their first emergency room visit were medication abuse (11.7%), followed by abdominal pain (9.8%) (Figure 1).

### 3.2. Emergency Medical Service Characteristics

The most common type of transportation of readmitted patients to the ED was through their own automobile. The type of transport was significantly different between the two groups (χ^2^ = 12.64, *p* = 0.005). In addition, according to the Korean Informative Classification of Diseases, the conditions that accounted for the greatest percentage in the readmission group after DAMA were abnormal laboratory findings (37.3%), followed by injury, poisoning, and other external causes (25.1%). Also, in the group that was not readmitted to the ED, injury, poisoning, and other external causes accounted for the highest percentage (37.6%), with a statistically significant difference (χ^2^ = 43.70, *p* < 0.001). There was no difference found between the two groups in terms of length of stay, the average of which was 5.52 ± 6.34 h (Table 2). 

The reasons for DAMA are shown in Table 2, and the reasons for requesting examination or treatment at other institutions (32.7%) and a financial burden (27.5%) accounted for the majority (Table 3).

### 3.3. Factors Influencing Readmission within 48 h after DAMA

Logistic regression analysis was performed using general and emergency medical service characteristics as independent variables to identify factors influencing readmissions after DAMA. Medical aid, foreign worker insurance, and automobile use as a means of transportation to the ED were found to be independent predictors of readmission. Compared to the national health insurance, the number of readmissions after DAMA was more than twice as likely in patients with medical aid and foreign worker insurance (OR (odds ratio) = 2.02, 95% CI (confidence interval): 1.46–2.83 and OR = 2.07, 95% CI: 1.04–4.09, respectively). In addition, compared to ambulance use, patients who arrived via automobile were almost 1.5 times more likely to be readmitted to the ED after DAMA (OR = 1.41, 95% CI: 1.08–1.82). However, age, gender, length of stay, and disease classification were not significantly associated with readmission (Table 4).

## 4. Discussion

This study aimed to identify the factors influencing readmission to the ED within 48 h after DAMA and provide data for establishing an intervention strategy to prevent complications, such as exacerbation of latent diseases, increase in readmission rates, and mortality. Factors found to influence readmission were having medical aid and foreign worker insurance as part of their health coverage and using an automobile as the means of transport to the ED. 

First, insurance-related factors were the biggest influencing factor on readmission after DAMA. The medical aid insurance type is speculated to have affected readmission after DAMA. The burden of medical expenses is somewhat lower than that of other insurance systems; therefore, it is considered to be a revisit. Being a recipient of medical aid in Korea refers to the need for medical protection according to the laws of the National Basic Living Security Act [15]. This applies to individuals whose four-person household income is less than 40% of the national average. These recipients are insured through the national fund, and the medical benefit is set at 400 days for each disease. Medical care patients generally have a low socioeconomic level; therefore, their disease management is not executed properly, leading to chronicity [16]. Consequently, it can be observed that there is a high possibility that symptoms of chronic diseases will reoccur after being voluntarily discharged from the hospital. Hence, there is a high probability of revisiting. It was found that most of the reasons for DAMA were to stop treatment and return home for economic reasons [10]. In this study, 27.5% of the patients, the second-highest rate among the causes of DAMA, chose voluntary discharge due to economic burden. Further research is needed as there is a lack of data on the use of medical institutions by medical aid recipients in Korea, and communication with consideration for various possibilities depending on the type of insurance is necessary for the clinical field. 

In addition, this study revealed that workers insured under the industrial accident insurance for foreigners were more likely to be admitted to the ED within 48 h of DAMA. In fact, the number of people prematurely discharged has been shown to be much higher among foreigners than among Koreans, and the short duration of stay is more likely due to financial rather than medical problems [6]. Moreover, there are a significant number of patients who delay medical treatment or are DAMA due to illegal stay, absence of insurance, or guardians [6]. Foreigners pay for medical expenses in the form of insurance depending on income, and the premiums are often relatively low because they do not have easily identified incomes or assets in Korea. From July 2019, all foreigners who have lived in Korea for more than 6 months must sign up for health insurance, receive the same health insurance benefits as Koreans, and pay insurance premiums accordingly [15]. The number of foreign patients DAMA may decrease in the future, as foreigners now have equal access. In addition, these results could be because the research institution is located in a neighborhood where automobile and ship manufacturing factories are densely populated, and injuries caused by industrial accidents are frequent among foreign workers. Therefore, multi-institutional follow-up studies on DAMA and readmission among foreigners are necessary.

The use of an automobile as the means of transportation to the hospital was shown to be a factor affecting readmission after DAMA. According to a 2019 survey by the National Statistical Office, 57.4% of patients presented to the hospital in vehicles [17]. In addition, if more than half of emergency room patients visit the hospital by private car, and emergency patients apply for an ambulance and cannot be dispatched to the desired emergency medical institution, it can be considered as using private transportation. In Korea, it is straightforward to use a taxi, especially as a means of transportation. Using a taxi or car is faster than waiting for an ambulance. In particular, since it is a revisit after DAMA, it is thought that using a private car can access the medical period faster than the first visit; therefore, it is speculated that many people use a private car. Since there are no previous studies on revisitation and visitation means after DAMA, it is difficult to compare and explain. Thus, further research is needed. 

In terms of age, the most frequent readmissions following DAMA were for patients between the ages of 50 to 60 years. Active seniors refer to those in their 50s and 60s who enjoy consumption, life, and leisure based on time and economic leisure [18]. They are interested in appearance and health care; therefore, it is a time to invest in themselves, and it can be observed as a result of continuous efforts for health and treatment. Therefore, it is speculated that emergency room medical staff need to pay more attention to the age groups 50s and 60s, that have high health requirements and are more likely to revisit the hospital even after DAMA.

Among the patients DAMA in this study, injuries, poisoning, and external causes accounted for a large percentage and was the second most common disease classification. These patients revisited the hospital within 24 h, and this is likely due to the research institution’s specialized center for accidents and trauma.

This study had some limitations. Due to its retrospective observational nature, there may have been potential confounding factors that could have influenced the exposure and outcome variables. Moreover, the study only included patients from a single institution, which calls into question the generalizability of the results.

Despite these limitations, this study is significant in the following points. This is the first study published to solve problems that may occur in DAMA patients by identifying factors affecting the early readmission of DAMA among patients who visited the emergency room of a tertiary hospital in Korea for 10 years.

## 5. Conclusions

The factors influencing early readmission after DAMA from ED included having medical aid and industrial accident insurance for foreign workers as their health insurance plan, as well as the use of an automobile as the means of transportation. ED healthcare providers should treat patients who are DAMA and those who are likely to return with a more careful and preventative management approach. Future studies should focus on strategies that can minimize the clinical, legal, and economic impact of DAMA.

## Figures and Tables

**Table 1:** Characteristics of study patients stratified by readmission within 48 h.

Variables	Total (*n* = 5445)	Readmission within 48 h		χ² or t (*p*)
		Yes (*n* = 351)	No (*n* = 5094)	
	*n* (%)	*n* (%)	*n* (%)	
Age (years), M ± SD	43.52 ± 22.12	44.42 ± 24.25	43.46 ± 21.96	−0.73 (0.467)
0–10	563 (10.3)	50 (14.2)	513 (10.1)	
11–20	294 (5.4)	11 (3.1)	283 (5.6)	
21–30	603 (11.1)	26 (7.4)	577 (11.3)	
31–40	786 (14.4)	46 (13.1)	740 (14.5)	
41–50	978 (18.0)	54 (15.4)	924 (18.1)	
51–60	1024 (18.8)	75 (21.4)	949 (18.6)	
61–70	576 (10.6)	46 (13.1)	530 (10.4)	
71–80	407 (7.5)	26 (7.4)	381 (7.5)	
≥81	214 (3.9)	17 (4.8)	197 (3.9)	
Gender				0.003 (0.957)
Male	3141 (57.7)	202 (57.5)	2939 (57.7)	
Female	2304 (42.3)	149 (42.5)	2155 (42.3)	
Insurance type				26.23 (< 0.001)
National health insurance	4496 (82.6)	275 (78.3)	4221 (82.9)	
Medical aid	444 (8.2)	49 (14.0)	395 (7.8)	
Employee insurance	113 (2.1)	7 (2.0)	106 (2.1)	
Private insurance	168 (3.1)	6 (1.7)	162 (3.2)	
Automobile insurance	142 (2.6)	4 (1.1)	138 (2.7)	
Foreign worker insurance	82 (1.5)	10 (2.8)	72 (1.4)	

Abbreviations: M, mean; SD, standard deviation.

**Table 2:** Characteristics of emergency medical services stratified by readmission within 48 h.

Variables	Total (*n* = 5445)	Readmission within 48 h		χ² or t (*p*)
		Yes (*n* = 351)	No (*n* = 5094)	
	*n* (%)	*n* (%)	*n* (%)	
Type of transportation to the ED				12.64 (0.005)
Ambulance	1959 (36.0)	99 (28.2)	1860 (36.5)	
Self-transport	3391 (62.3)	249 (71.0)	3142 (61.6)	
Inter-facility transfer	95 (1.7)	3 (0.9)	92 (1.8)	
Length of stay in the ED (h), M ± SD	5.52 ± 6.34	5.56 ± 5.30	5.52 ± 6.40	−0.12 (0.903)
Korea Informative Classification of Diseases				43.70 (<0.001)
Infectious diseases	58 (1.1)	5 (1.4)	53 (1.0)	
Neoplasms	94 (1.7)	13 (3.7)	81 (1.6)	
Hematology, endocrine system	113 (2.1)	8 (2.3)	105 (2.1)	
Mental and behavioral disorders	396 (7.3)	25 (7.1)	371 (7.3)	
Nervous system	115 (2.1)	8 (2.3)	107 (2.1)	
Eye and adnexa	43 (0.8)	1 (0.3)	42 (0.8)	
Circulatory system	147 (2.7)	18 (5.1)	129 (2.5)	
Respiratory system	116 (2.1)	8 (2.3)	108 (2.1)	
Digestive system	265 (4.9)	22 (6.3)	243 (4.8)	
Skin and musculoskeletal system	141 (2.6)	12 (3.4)	129 (2.5)	
Genitourinary system, pregnancy, congenital malformations	88 (1.6)	12 (3.4)	76 (1.5)	
Abnormal laboratory findings	1864 (34.2)	131 (37.3)	1733 (34.0)	
Injury, poisoning, and certain other consequences of external causes	2005 (36.8)	88 (25.1)	1917 (37.6)	

Abbreviations: ED, emergency department; M, mean; SD, standard deviation.

**Table 3:** The reason for discharge against medical advice (*n* = 5445).

Reason	N	(%)
Want additional treatment at other hospitals	1781	32.7
Economic burden for treatment and evaluation	1497	27.5
Want to observe progress at home	915	9.2
Lack of awareness of the patient’s severity	414	7.6
Distrust of the health providers	337	6.2

**Table 4:** Factors influencing readmission within 48 h after AMA discharge.

Variable	OR	95% Cl	*p*-Value
Age	1.00	0.95–1.01	0.964
Gender Female (REF: Male)	1.02	0.82–1.28	0.866
Medical insurance status (REF: National health insurance)			
Medical aid	2.02	1.46–2.83	<0.001
The employee insured	1.38	0.62–3.08	0.429
Private insurance	0.83	0.36–1.93	0.672
Automobile insurance	0.69	0.25–1.92	0.480
Foreign worker insurance	2.07	1.04–4.09	0.037
Type of transportation to ED (REF: Ambulance)			
Self-transport	1.41	1.08–1.82	0.010
Inter-facility transfer	0.56	0.17–1.81	0.332
Length of stay in the ED (hour)	1.00	0.99–1.00	0.867
Korea Informative Classification of Diseases (REF: Infectious diseases)			
Neoplasms	1.67	0.56–5.03	0.359
Hematology, endocrine system	0.79	0.24–2.57	0.696
Mental and behavioral disorders	0.76	0.28–2.09	0.591
Nervous system	0.82	0.25–2.63	0.732
Eye and adnexa	0.22	0.02–1.92	0.169
Circulatory system	1.41	0.49–4.06	0.529
Respiratory system	0.74	0.23–2.34	0.621
Digestive system	0.90	0.32–2.50	0.838
Skin and musculoskeletal system	0.93	0.31–2.80	0.898
Genitourinary system, pregnancy, congenital malformations	1.68	0.55–5.08	0.361
Abnormal clinical and laboratory findings	0.81	0.32–2.07	0.657
Injury, poisoning, and certain other consequences of external causes	0.54	0.21–1.40	0.200

Abbreviations: CI, confidence interval; ED, emergency department; OR, odds ratio.

## Data Availability

The data presented in this study are available on request from the corresponding author.
